# The dataset describes: HIF-1 α expression and LPS mediated cytokine production in MKP-1 deficient bone marrow derived murine macrophages

**DOI:** 10.1016/j.dib.2017.07.036

**Published:** 2017-07-20

**Authors:** Harvinder Talwar, Christian Bauerfeld, Yusen Liu, Lobelia Samavati

**Affiliations:** a Department of Medicine, Division of Pulmonary, Critical Care and Sleep Medicine, Wayne State University School of Medicine and Detroit Medical Center, Detroit, MI 48201, United States; b Department of Pediatrics, Division of Critical Care, Wayne State University School of Medicine and ,Children's Hospital of Michigan Detroit, MI 48201, United States; c Center for Perinatal Research, The Research Institute at Nationwide Children's Hospital, Columbus, OH 43205, United States; d Center for Molecular Medicine and Genetics, Wayne State University School of Medicine, Detroit, MI 48201, United States

**Keywords:** MKP-1, HIF-1α, p300, IL-1β, Bone marrow derived macrophages

## Abstract

The data presented in this article are related to the research article entitled “MKP-1 negatively regulates LPS-mediated IL-1β production through p38 activation and HIF-1α expression” (Talwar et al., 2017) [1]. This data describes that LPS-mediated p38 and JNK phosphorylation is enhanced in MKP-1 deficient macrophages. HIF-1α expression and its nuclear accumulation are significantly increased in the nuclear extracts of MKP-1 deficient BMDMs. MKP-1 deficient BMDMs exhibited higher expression of the coactivator p300 of HIF-1α both at baseline and after LPS challenge. Inhibition of p38 MAP kinase decreased LPS mediated HIF-1α protein levels and its nuclear translocation in MKP-1 deficient BMDMs. Inhibition of p38 MAP kinase inhibited LPS induced pro-inflammatory cytokines including IL-1β, IL-6 and TNF-α.

**Specifications Table**

**Table:** 

Subject area	Immunology and Inflammation
More specific	Cell signaling
subject area	
Type of data	Figures
How data was acquired	Protein extraction and immunoblotting.
	Enzyme linked immunoabsorbent assay (ELISA) for cytokine determination. Nuclear extraction
Data format	Analyzed
Experimental factors	Bone marrow derived macrophages (BMDMs) were prepared from WT and MKP-1deficient mice.
Experimental features	Western blot analyses were performed on cell lysates and nuclear fractions obtained from WT and MKP-1 deficient mice.
	Cytokines released into cell culture media were analyzed by ELISA.
Data source	
location	Wayne State University, Detroit, Michigan, USA.
Data accessibility	The data is with the article.

**Value of Data**

• This data shows an enhanced expression of HIF-1α in MKP-1 deficient macrophages [1].
• Nuclear extracts of MKP-1 deficient macrophages exhibited higher accumulation of HIF-1α along with its transcriptional co-activator p300 [2], [3] as compared to WT macrophages.
• Presented data has the potential to guide future exploration of the mechanisms involved in the stabilization of HIF-1α in absence of MKP-1or through increased activation of p38 MAPK.

## 1 Data

**Fig. 1:**
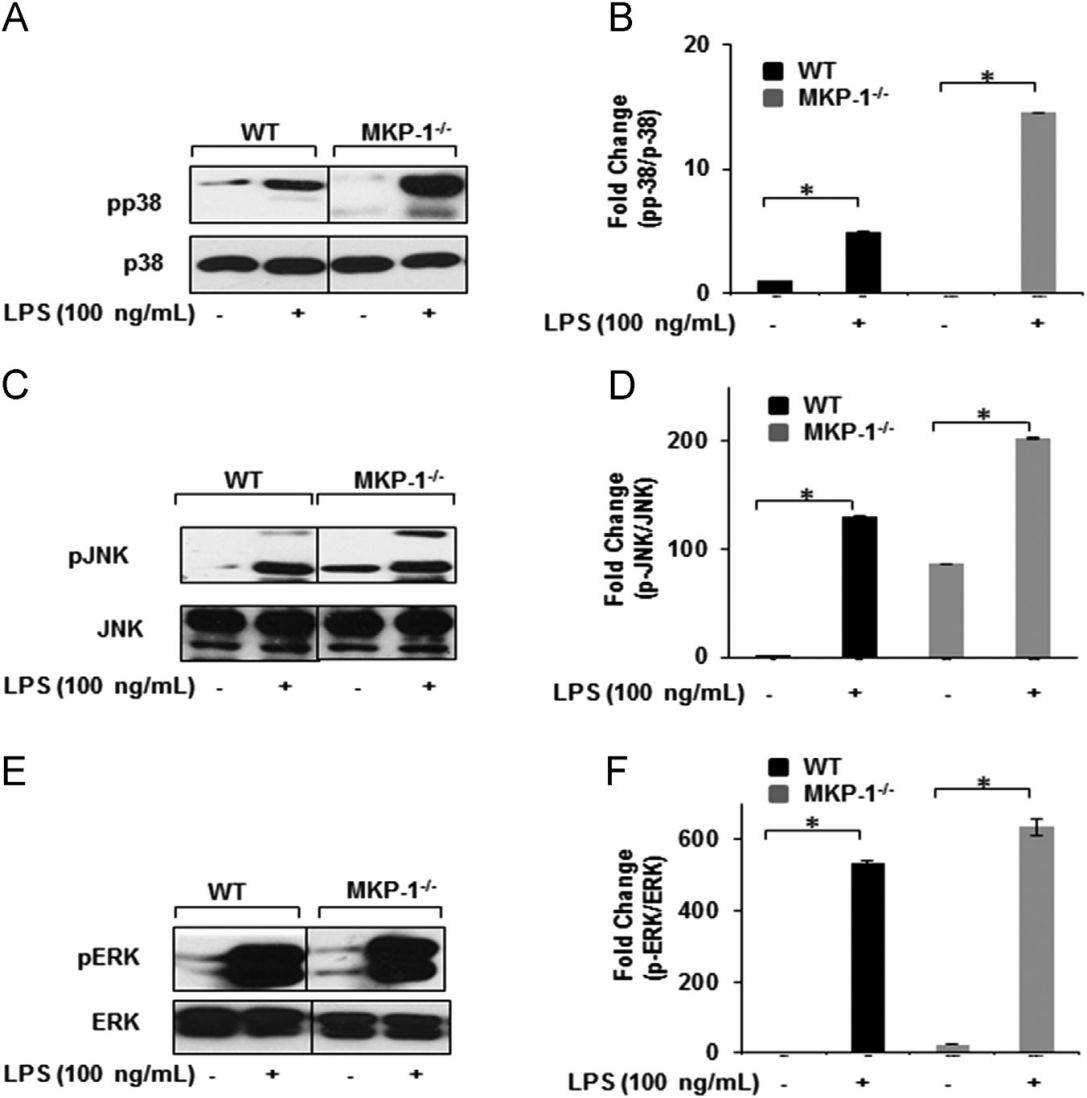
MKP-1^-/-^ BMDMs exhibit higher p38 and JNK activation in response to LPS. BMDMs derived from WT and MKP-1^-/-^ mice were cultured and challenged with LPS (100 ng/mL) for 30 min. Whole cell extracts were prepared and subjected to SDS-PAGE and Western blot analysis using phospho-specific antibodies against p38 (Thr180/Tyr182), ERK (Thr202/Tyr204) and JNK (Thr183/Tyr185). Equal loading was confirmed using the corresponding non-phosphorylated antibodies. (A) MKP-1^-/-^ BMDMs exhibited higher activation of pp38 by LPS as compared to WT. (B) Densitometric values expressed as fold changes of the ratio of phosphorylated p38/total p38. (C) MKP-1^-/-^ BMDMs exhibited higher phospho-JNK at baseline with an increase in phosphorylation in response to LPS challenge. (D) Densitometric values expressed as fold increase of the ratio of phosphorylated JNK (54 kDa and 46 kDa)/total JNK. (E) Phosphorylation of ERK after LPS stimulation in WT and MKP-1^-/-^ BMDMs. (F) Densitometric values expressed as fold increase of the ratio of phosphorylated ERK/total ERK. All densitometric data represent mean±SEM of at least 4 independent experiments. * Represents a *p* value<0.05.

**Fig. 2:**
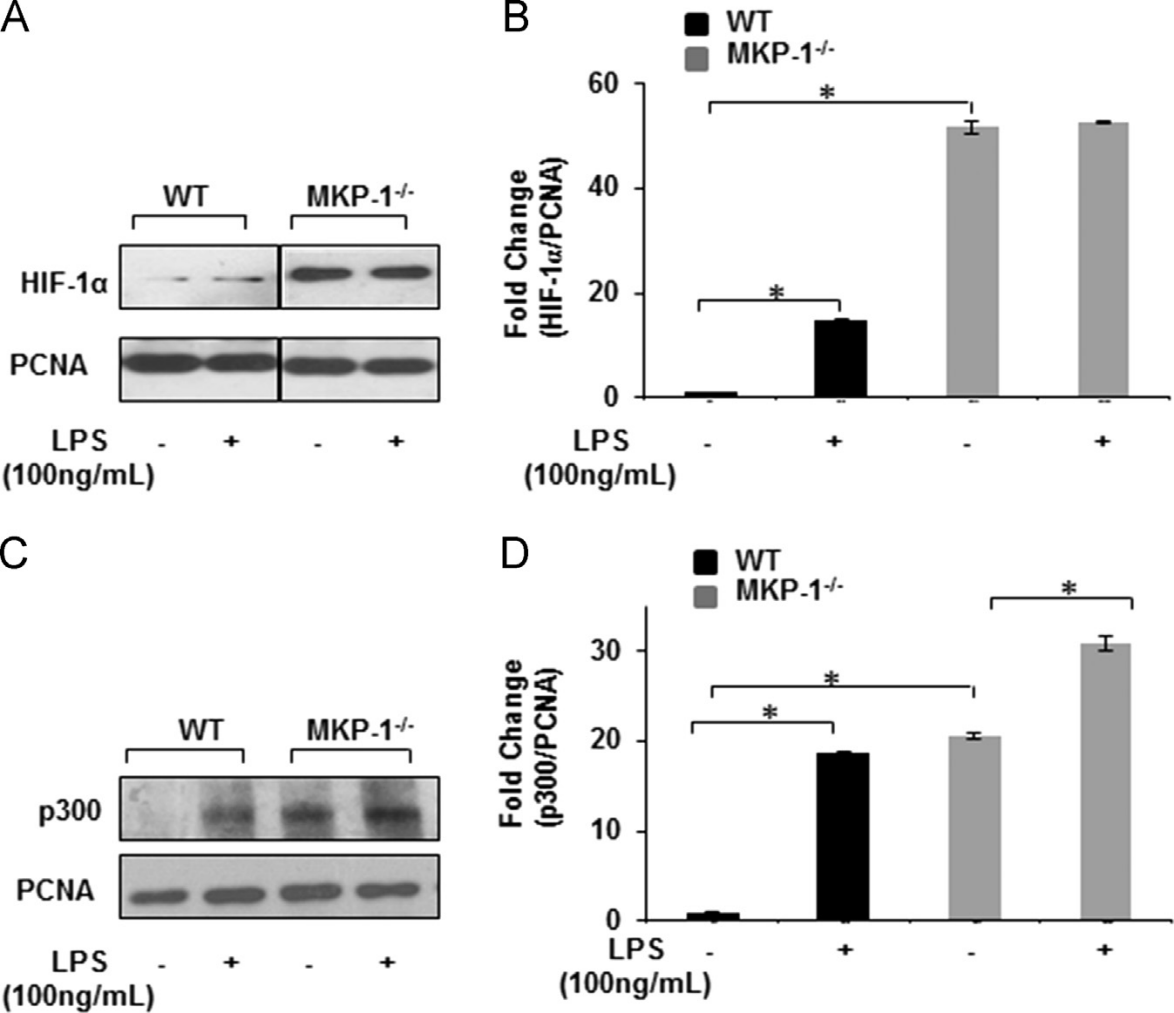
MKP-1^-/-^ BMDMs exhibit higher HIF-1α and p300 expression in nuclear extracts: WT and MKP-1 deficient macrophages were cultured and challenged with LPS (100 ng/mL) for 3 h. Nuclear extracts were prepared and subjected to SDS-PAGE. Western blot analysis was performed using specific antibodies against HIF-1α (A) and p300 (C). Equal loading was confirmed using antibody against proliferating cell nuclear antigen (PCNA). Densitometric values expressed as fold change of the ratio of HIF-1α /PCNA (B) and p300/PCNA (D). Data represent mean±SEM of at least 3 independent experiments. * Represents a *p* value<0.05. MKP-1 deficient macrophages show an increased translocation of HIF-1α and p300 to the nucleus as compared to WT.

**Fig. 3:**
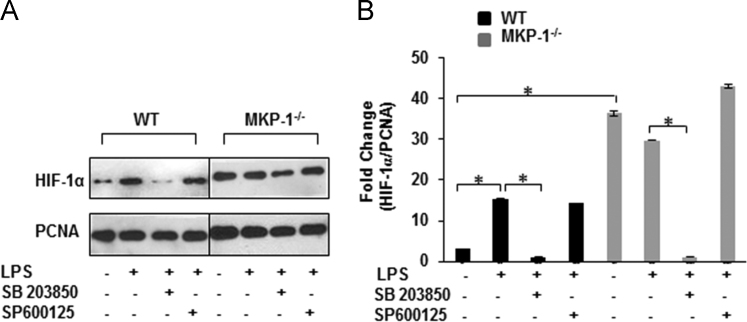
SB203850 inhibits LPS-induced HIF-1α nuclear accumulation: (A) WT and MKP-1 deficient macrophages were cultured and challenged with LPS (100 ng/mL) for 3 h in the presence and absence of SB 203850 (10 µM), a specific inhibitor of p38 and SP 600125 (20 µM), a specific inhibitor of JNK. Nuclear extracts were prepared and subjected to SDS-PAGE. Western blot analysis was performed using specific antibodies against HIF-1α. Equal loading was confirmed with PCNA. MKP-1 deficient macrophages show a decreased translocation of HIF-1α to the nucleus after specific inhibition of p38 but not JNK. (B) Densitometric values expressed as fold change of the ratio of HIF-1α/PCNA. Data represents mean±SEM of at least 3 experiments. * Represents a *p* value<0.05.

**Fig. 4:**
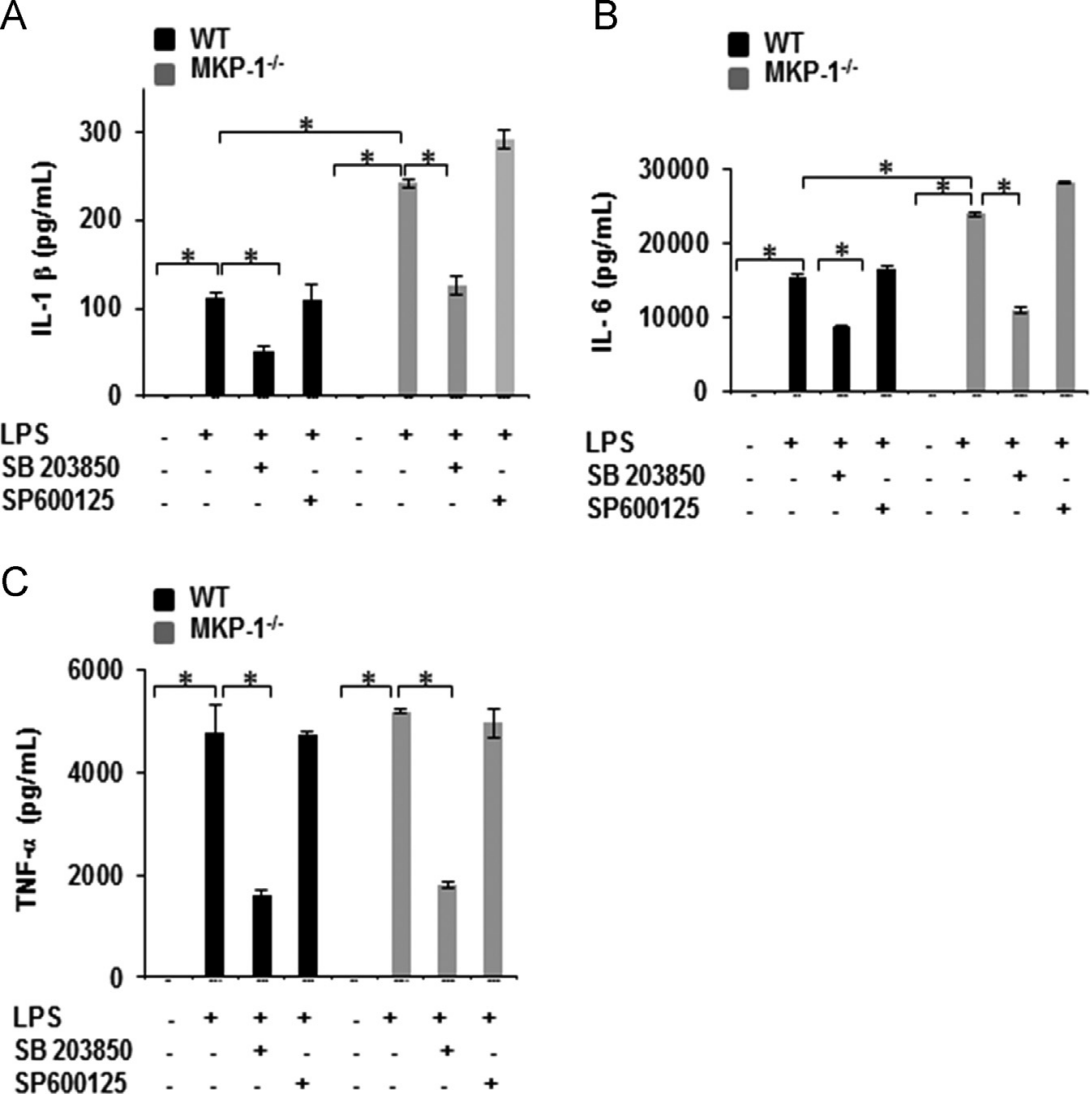
Inhibition of p38 MAP kinase blocks LPS induced IL-1β, IL-6, and TNF-α production in WT and MKP-1 deficient BMDMs: BMDMs derived from WT and MKP-1^-/-^ mice were treated with LPS (100 ng/mL) for 24 h in the presence and absence of SB 203850 (specific inhibitor of p38) and SP 600125 (specific inhibitor of JNK). Conditioned media were analyzed via *ELISA* for IL-1β (1:1 sample dilution) (A), IL-6 (1:40 sample dilution) (B), and TNF-α (1:20 sample dilution) (C). Data are presented as mean±SEM (*n*=3) *p*<0.05. Production of IL-1β, IL-6 and TNF-α were decreased by inhibition of p38 but not JNK.

This dataset extends the reproducibility of the recent published data [1]. BMDMs derived from WT and MKP-1^-/-^ mice exhibit higher p38 and JNK activation in response to LPS (Fig. 1). HIF-1α expression is significantly increased at baseline and in response to LPS challenge in nuclear extracts of MKP-1 deficient BMDMs under normoxic condition as compared to WT BMDMs (Fig. 2). In nuclear extracts of MKP-1 deficient BMDMs expression of p300, a transcriptional coactivator that binds with HIF-1α at the promoter regions of targeted genes, is significantly increased at baseline and in response to LPS treatment (Fig. 2). LPS-induced HIF-1α expression is significantly inhibited by SB203850, a specific inhibitor of p38 but not by SP600125, a specific inhibitor of JNK (Fig. 3) LPS-induced IL-1β, TNF-α, and IL-6 production in MKP-1 deficient BMDMs are significantly inhibited by SB203850, a specific inhibitor of p38 MAPK (Fig. 4).

## 2 Experimental design, materials and methods

### 2.1 Chemicals and antibodies

LPS was purchased from Invivogen (San Diego, CA). Phospho-specific antibodies against the phosphorylated form of ERK1/2, p38, JNK, as well as total ERK1/2, JNK, and β-actin were purchased from Cell Signaling Technology (Beverly, MA). Total p38 antibody was purchased from Santa Cruz Biotechnology (Santa Cruz, CA). The IL-1β antibody was purchased from R&D Systems (Minneapolis, MN). The HIF-1α antibody was purchased from Bioss Inc (Woburn, MA). Horseradish peroxidase (HRP)-conjugated anti-mouse and anti-rabbit IgG secondary antibodies were purchased from Cell Signaling Technology, and horseradish peroxidase (HRP)-conjugated anti-goat antibody was purchased from Santa Cruz Biotechnology.

### 2.2 Mice and isolation of bone marrow derived macrophages (BMDMs)

Wild-type (WT), MKP-1 knockout mice were generated as previously described [4]. Animal studies were approved by the Institutional Committee on Animal Use and Care of the Research Institute at Nationwide Children's Hospital. BMDMs from mice were prepared as described previously [5]. Briefly, femurs and tibias from 6- to 12-week-old mice were dissected and the bone marrow was flushed out. Macrophages were cultured with IMDM media containing glutamine, sodium pyruvate, 10% heat-inactivated fetal FBS, 30% L929 conditioned medium, and antibiotics for 5–7 days. BMDMs were re-plated at a density of 2×10^6^ cells/well the day before the experiment.

### 2.3 Protein extraction and immunoblotting

After the appropriate treatments, cells were washed with PBS, and harvested in RIPA buffer (Millipore, Billerica, MA) containing protease inhibitor and anti-phosphatase cocktails, as previously described [6]. Equal amounts of proteins (15 μg) were mixed with the same volume of 2x sample buffer, separated on 10% SDS-polyacrylamide gel electrophoresis and transferred to a polyvinylidene di-fluoride (PVDF) membrane (Bio-Rad, Hercules, CA) at 18 V for 1 h using a semi dry transfer cell (Bio-Rad) as previously described [3]. The PVDF membrane was blocked with 5% dry milk in TBST (Tris-buffered saline with 0.1% Tween-20), rinsed, and incubated with primary antibody overnight. The blots were washed and incubated with HRP-conjugated secondary anti-IgG antibody. Membranes were washed and immunoreactive bands were visualized using a chemiluminescent substrate (ECL-Plus, GE Healthcare, Pittsburgh, PA). Images were captured on Hyblot CL film (Denville Scientific Inc, Metuchen, NJ). Optical density analysis of signals was performed using ImageQuant software (version 5, GE Healthcare).

### 2.4 Enzyme linked immunosorbent assay (ELISA)

IL-1β, Il-6, and TNF-α cytokine levels in cell culture supernatants were measured using ELISA DuoKits (R&D Systems) as previously described [1], [7].

### 2.5 Fractionation of cytoplasmic and nuclear proteins

The cytoplasmic and nuclear fractions were separated as described previously [6]. Briefly, after treatment the cells were resuspended in a hypotonic buffer (10 mm HEPES, pH 7.9, 0.5% Igepal, 2 mm MgCl_2_, 10 mm KCl, 0.1 mm EDTA, 0.5 mm phenylmethylsulfonyl fluoride, 1.0 μg/ml leupeptin, and 1.0 μg/ml aprotinin) and incubated on ice for 10 min. After centrifugation at 14,000×*g* for 1 min at 4 °C, the supernatant (cytoplasmic) and the pellets (nuclear fraction) were collected.
