# Supplementary material for: The dataset describes: HIF-1 α expression and LPS mediated cytokine production in MKP-1 deficient bone marrow derived murine macrophages
Source: Data Brief. 2017 Jul 20;14:56–61. doi: 10.1016/j.dib.2017.07.036 (PMC5526519; doi:10.1016/j.dib.2017.07.036)
Supplement: Supplementary file 1 — Supplementary material [file mmc1.docx]

Authors declare no conflict of interests.
